# The Compound Terminalia Chebula Extract Alleviates PEDV-Induced Colonic Injury in Suckling Piglets by Enhancing Antioxidant Capacity, Suppressing Inflammation, Restoring Intestinal Function, and Inhibiting Viral Replication

**DOI:** 10.3390/ani16132085

**Published:** 2026-07-06

**Authors:** Yanyan Zhang, Lingling Gan, Muzi Li, Jiaxing Wang, Zongyun Li, Zhonghua Li, Lei Wang, Di Zhao, Tao Wu, Dan Yi, Yongqing Hou

**Affiliations:** 1 Hubei Key Laboratory of Animal Nutrition and Feed Science, Wuhan Polytechnic University, Wuhan 430023, China; 2 Engineering Research Center of Feed Protein Resources of Agricultural By-Products, Ministry of Education, Wuhan Polytechnic University, Wuhan 430023, China

**Keywords:** compound terminalia chebula extract, piglets, PEDV, colonic injury, crypt depth

## Abstract

The protective effect of Compound terminalia chebula extract (HL) against colonic injury induced by Porcine epidemic diarrhea virus (PEDV) infection in neonatal piglets remains unclear. This study aimed to evaluate the mitigating effects of HL on PEDV-induced colonic injury and elucidate the underlying mechanisms. Eighteen 7-day-old Duroc × Landrace × Large White piglets (2.58 ± 0.05 kg) were randomly assigned to three groups (n = 6/group): CON (blank control), PEDV (infected), and HL + PEDV (HL-supplemented + infected). The 11-day trial included 3 days of acclimatization (days 0–3) and an 8-day experimental period (days 4–11). HL (10 mg/kg BW) was orally administered daily to the HL + PEDV group. On day 8, PEDV and HL + PEDV groups were challenged with 3 mL PEDV (3 × 10^6^ TCID_50_/mL), while CON received Dulbecco’s Modified Eagle Medium (DMEM). All piglets were euthanized on day 11 for colonic tissue collection. Results indicated that PEDV infection induced colonic injury, manifested by a significant increase in crypt depth and disruption of intestinal homeostasis. This was evidenced by impaired barrier integrity (upregulation of matrix metalloproteinase-7 gene [*MMP7*] and matrix metalloproteinase 13 gene [*MMP13*], mucus disorganization (elevation of mucin 5AC gene [*MUC5AC*]), oxidative stress (reduced catalase [CAT] activity and increased malondialdehyde [MDA] levels in serum and colon), and inflammation (upregulation of regenerative islet-derived protein 3γ gene [*REG3G*], S100 calcium-binding protein A8/A9 gene [*S100A8/A9*], and interleukin-1β gene [*IL-1β*]). Additionally, PEDV impaired colonic ion transport by downregulating calcium channel genes (Transient Receptor Potential Cation Channel Subfamily V Member 6 gene [*TRPV6*], Transient Receptor Potential Cation Channel Subfamily M Member 6 gene [*TRPM6*]). Notably, HL supplementation effectively reversed these adverse effects. HL restored colonic morphology, increased CAT activity, reduced MDA accumulation, and suppressed inflammatory gene expression. Furthermore, HL modulated the expression of genes involved in water and ion transport upregulating Aquaporin 7 gene (*AQP7*), Chloride Channel Accessory 4 gene (*CLCA4*), Sodium-Hydrogen Exchanger 3 gene (*NHE3*), Transient Receptor Potential Vanilloid 6 (*TRPV6*), and Transient Receptor Potential Melastatin 6 gene (*TRPM6*) and significantly inhibited PEDV replication, as indicated by the downregulation of the transcription levels of PEDV membranegene (*M*), nucleocapsid gene (*N*), and spike gene (*S*). Taken together, HL alleviates PEDV-triggered colonic tissue damage in suckling piglets via improving colonic antioxidant capacity, mitigating inflammatory response, partially regulating intestinal barrier and ion/water transport-related genes, and downregulating the transcription of PEDV structural genes at molecular and histological levels.

## Simple Summary

The colon maintains critical intestinal homeostasis by regulating water and electrolyte absorption, mucosal barrier integrity, and intestinal immune responses in suckling piglets. Viral intestinal damage caused by porcine epidemic diarrhea virus can impair colon structure and function, leading to severe diarrhea and higher piglet mortality and resulting in considerable economic losses in global pig farming. Suitable nutritional strategies to relieve such virus-induced intestinal damage remain insufficient. This study explored the protective effects of compound Terminalia chebula extract on colon injury in suckling piglets challenged with porcine epidemic diarrhea virus and analyzed its potential regulatory characteristics. We detected intestinal tissue morphology, antioxidant status, inflammatory response, ion transport function, and viral proliferation levels among normal piglets, virus-infected piglets, and extract-treated piglets. The viral infection damaged intestinal structure, weakened antioxidant capacity, triggered excessive inflammation, and disrupted water and ion balance, thereby facilitating viral replication. In comparison, the plant extract administration alleviated colon tissue lesions, reduced oxidative and inflammatory damage, restored intestinal transport balance, and inhibited viral proliferation. These findings deepen the understanding of the protective mechanism of natural plant extracts against viral intestinal injury and provide a feasible nutritional strategy to protect piglet intestinal health and reduce viral diarrhea losses in pig production.

## 1. Introduction

The intestine serves as the primary site for nutrient absorption and a critical barrier against pathogen invasion in neonatal piglets. Its structural integrity is a pivotal determinant of growth performance and disease resistance, with disruption by weaning stress having substantial impacts on the global swine industry [1]. However, the intestinal mucosa of suckling piglets remains physiologically immature, characterized by an underdeveloped barrier system and naive immune defenses. This renders them highly susceptible to debilitating enteric pathogens, among which Porcine Epidemic Diarrhea Virus (PEDV) is the most prevalent and economically detrimental [2,3].

PEDV is an enveloped, positive-sense, single-stranded RNA virus belonging to the genus *Alphacoronavirus* within the family *Coronaviridae* [4]. The virus is highly contagious and primarily transmitted via the fecal–oral route, causing porcine epidemic diarrhea (PED), a disease characterized by acute watery diarrhea, vomiting, and severe dehydration [3]. PEDV specifically targets and destroys intestinal epithelial cells, leading to villous atrophy, crypt hyperplasia, and malabsorption syndrome [5]. While the pathology of PEDV in the small intestine is well-documented, emerging evidence indicates that the virus also compromises the structural integrity of the colon, disrupts mucosal barrier function, and alters microbial homeostasis, contributing to systemic illness [6]. Neonatal piglets (<3 days old) that fail to acquire adequate maternal antibodies suffer the most severe consequences, with mortality rates frequently approaching 100%. Despite the availability of commercial vaccines, the continuous evolution and mutation of PEDV strains have diminished cross-protective efficacy. Currently, specific antiviral therapies are scarce, and conventional control strategies are insufficient to prevent viral-induced intestinal damage, underscoring the urgent need for novel prophylactic and therapeutic interventions [7].

Following the global trend towards antibiotic restriction in animal husbandry, there is an escalating demand for safe, natural, and multifunctional alternatives. Plant-derived extracts have garnered significant attention due to their antioxidant, anti-inflammatory, and direct antiviral properties. *Terminalia chebula* Retz. (Combretaceae), known as Chebulic Myrobalan, is a medicinal fruit traditionally used to treat diarrhea and gastrointestinal disorders. Modern phytochemistry reveals that it is rich in hydrolyzable tannins (e.g., chebulic acid, corilagin) and phenolic acids, which contribute to its broad-spectrum bioactivities [8]. Tannins from *Terminalia chebula* can inhibit coronavirus entry and proteolytic processing. Studies have demonstrated that chebulinic acid suppresses porcine epidemic diarrhea virus (PEDV) infection by inhibiting viral entry and main protease [9], while molecular docking suggests potential inhibitory effects on SARS-CoV-2 proteins [10]. Furthermore, its extracts exhibit potent anti-inflammatory effects by suppressing the NF-κB and MAPK signaling pathways, thereby protecting endothelial and epithelial barrier functions [11]. In the context of intestinal health, *Terminalia chebula* has been shown to alleviate dextran sulfate sodium (DSS)-induced ulcerative colitis in murine models and improve intestinal morphology and diarrhea incidence in weaned piglets [12]. Chebulic myrobalan extract can replace zinc oxide, enhance the antioxidant and anti-inflammatory capabilities of weaned piglets, improve growth performance, and maintain gut health [13]. These findings suggest its potential as a functional feed additive to mitigate enteric disorders.

However, despite these promising pharmacological profiles, the protective effect of HL against PEDV-induced colonic injury in vivo remains unexplored. Given the colon’s vital role in water absorption and immune homeostasis, elucidating whether HL can restore colonic barrier integrity and suppress viral replication is of great scientific interest. Therefore, this study aimed to systematically evaluate the protective effects of a compound HL (standardized to contain ≥ 40% chebulic acid) on PEDV-induced colonic damage in a suckling piglet model. We hypothesized that HL would alleviate colonic injury by enhancing antioxidant capacity, suppressing inflammatory responses, restoring ion transport function, and inhibiting viral replication.

## 2. Materials and Methods

### 2.1. Experimental Materials

The Yunnan strain of Porcine Epidemic Diarrhea Virus (PEDV) was obtained from the Hubei Provincial Key Laboratory of Animal Nutrition and Feed Science (Wuhan, Hubei, China). The virus was propagated in Vero cells, and the stock suspension had a titer of 1 × 10^6^ TCID_50_/mL. The compound *Terminalia chebula* extract (HL, containing ≥ 40% active ingredients such as chebulic acid) was provided by Hubei Haohua Biotechnology Co., Ltd. (Wuhan, China). For administration, HL was dissolved in sterile saline to achieve the desired concentration. The milk replacer consisted of reconstituted whole milk powder (Nouriz, New Zealand), prepared according to the manufacturer’s instructions.

### 2.2. Experimental Animals and Design

All animal procedures were approved by the Institutional Animal Ethics Committee of Wuhan Polytechnic University (Approval No. WPU202405006). The euthanasia of suckling piglets strictly followed the humane requirements specified in this study. Eighteen healthy, 7-day-old Duroc × Landrace × Large White crossbred suckling piglets (initial body weight: 2.58 ± 0.05 kg; 9 males and 9 females) were sourced from a PEDV-negative commercial farm (Hubei Xufeng Agricultural Development Co., Ltd., Wuhan, China). Piglets were blocked by body weight and sex, and then randomly assigned to one of three treatment groups (n = 6 per group): the Control group (CON), which received no virus or extract treatment; the PEDV-infected group (PEDV), which was challenged with PEDV only; and the HL + PEDV group (HL + PEDV), which was administered HL extract prior to the PEDV challenge.

Restricted by experimental feeding space, breeding funds and animal supply, we cannot expand the sample quantity in the current trial. The limited sample size leads to insufficient statistical power to analyze sex main effects and treatment-sex interactions, so AVMA Guidelines for the Euthanasia of Animals (2020 Edition) relevant statistical analyses were not performed in this study. Piglets were housed in environmentally controlled isolator units with ad libitum access to water. The ambient temperature was maintained at 28 ± 2 °C, with a 12 h light/dark cycle. The experiment spanned 11 days. Days 0–3 constituted an acclimatization period. From days 4 to 10, piglets in the HL + PEDV group received a daily oral gavage of HL at a dose of 10 mg/kg body weight (BW) (dissolved in 2 mL of milk), while piglets in the CON and PEDV groups received an equivalent volume of milk vehicle. On day 8, piglets in the PEDV and HL + PEDV groups were orally inoculated with 3 mL of PEDV suspension (10^6^ TCID_50_/mL). The CON group received 3 mL of sterile Modified Eagle’s Medium (DMEM) as a mock control. To ensure accurate sampling and minimize stress, all piglets were deprived of feed and water from 22:00 on day 10. On the morning of day 11, anesthesia was induced via intramuscular injection of Zoletil 50 (Virbac, Carros, France) (10 mg/kg BW). Following loss of consciousness and confirmation of deep anesthesia (absence of pedal reflex), euthanasia was performed via exsanguination, and colonic tissue samples were immediately collected. Samples designated for histological analysis were fixed in 4% paraformaldehyde, whereas those for molecular assays were placed in cryogenic tubes, snap-frozen in liquid nitrogen and stored at −80 °C until analysis. Colonic tissue samples prepared for qPCR and molecular biochemical tests were slowly thawed at 4 °C to maintain RNA and protein integrity throughout the pretreatment process. Subsequently, total RNA was extracted from colon tissues for molecular analysis.

### 2.3. Rearing Management

Piglets in each experimental group were housed in separate pens with physical barriers to prevent direct contact and minimize the risk of cross-infection. A single suckling piglet was defined as an independent experimental unit. Although piglets of the same treatment group were raised in one pen, each piglet had independent feeding space and no cross interference between individuals from different treatments. Each piglet was regarded as a biologically independent replicate to avoid pseudoreplication bias. A fresh air purification system was operational throughout the trial to ensure air circulation and cleanliness; the filtration level complied with laboratory animal standards, and units were cleaned regularly to maintain ventilation efficiency. The ambient temperature was strictly maintained at 30 °C ± 1 °C, and relative humidity was kept at 60% using coordinated humidification, dehumidification, and ventilation controls to provide a thermoneutral environment.

A timed, measured feeding regimen was implemented with five daily feedings at 07:30, 11:00, 15:00, 18:00, and 21:00. Piglets were fed an equal volume of reconstituted milk (prepared by mixing milk powder with water at a 1:5 mass-to-volume ratio; 20 g milk powder per piglet per meal). Fresh drinking water was provided immediately after the morning, afternoon, and evening feedings (07:30, 15:00, and 21:00) and was maintained at an appropriate temperature to prevent intestinal irritation. The watering system, constructed of sterile materials, was cleaned and disinfected daily to ensure hygiene and meet physiological requirements. Additionally, feeding behavior and clinical signs were meticulously observed and recorded before and after each feeding.

### 2.4. Sample Collection and Processing

On day 11 of the experiment, piglets were anesthetized via intramuscular injection of Zoletil 50 and subsequently euthanized. The mid-colon segment was excised and flushed gently with ice-cold phosphate-buffered saline (PBS) to remove luminal contents. A 2–3 cm portion of this segment was immediately fixed in pre-chilled 4% paraformaldehyde for histological section preparation. The remaining colonic tissue was finely minced on an ice-cold surface, wrapped in aluminum foil, labeled, and snap-frozen in liquid nitrogen for subsequent storage at −80 °C.

### 2.5. Morphological Analysis of Colonic Tissues

Colonic tissue sections were prepared as paraffin-embedded samples and stained with hematoxylin and eosin (H&E) by Wuhan Bolofu Biotechnology Co., Ltd. (Wuhan, China). Morphological assessment was performed using an Olympus BX-41TF light microscope (Olympus Corporation, Tokyo, Japan). Five intact, well-oriented crypt-villus units per section were randomly selected. Crypt depth (CD) was measured in randomly selected, intact, and well-oriented crypt units from each tissue section. All measurements were conducted using CellSens Dimension 1.18 software (Olympus (Olympus Corporation, Tokyo, Japan).

### 2.6. Biochemical Assay of Antioxidant Status

To evaluate the redox state in the colonic mucosa, tissue samples were homogenized in ice-cold physiological saline (0.9% NaCl) and centrifuged at 3000× *g* for 10 min at 4 °C. The supernatants were collected for subsequent analysis. Protein concentration was determined using the BCA assay kit. The activities of antioxidant enzymes, including glutathione peroxidase (GSH-Px), total superoxide dismutase (T-SOD), and catalase (CAT), were quantified using commercially available assay kits (Nanjing Jiancheng Bioengineering Institute, Nanjing, China). Additionally, the levels of oxidative stress markers, such as myeloperoxidase (MPO), malondialdehyde (MDA), and hydrogen peroxide (H_2_O_2_), were determined spectrophotometrically according to the manufacturer’s instructions. All values were normalized to the total protein content to ensure data accuracy.

### 2.7. Quantitative Real-Time PCR Analysis

Colon tissue preserved in liquid nitrogen was ground and crushed under liquid nitrogen conditions, and 100 mg of tissue sample was accurately weighed. Total RNA was isolated using the RNAiso Plus Kit (TaKaRa, Dalian, China). Complementary DNA (cDNA) was synthesized via reverse transcription with the PrimeScript™ RT Reverse Transcription Kit containing gDNA Eraser (TaKaRa, Dalian, China). The reaction system was prepared using the SYBR^®^ Premix Ex Taq™ (Tli RNaseH Plus) Kit (TaKaRa, Dalian, China), and quantitative real-time PCR (qPCR) was conducted on a 7500 Rapid Real-Time PCR System (Applied Biosystems, Foster City, CA, USA). Ribosomal protein L19 (*RPL19*) was used as the internal reference gene in this study. The 2^−∆∆Ct^ method was applied to calculate the relative gene expression levels of each group [14], and the detailed primer sequences are shown in Table 1.

### 2.8. Statistical Analysis

All data were analyzed using SPSS Statistics software (version 26.0; IBM Corporation, Armonk, NY, USA). Differences among experimental groups were assessed by one-way analysis of variance (ANOVA), followed by Tukey’s multiple comparison test. Data are presented as the mean ± standard error of the mean (SEM). The probability value of *p* < 0.05 was considered statistically significant.

## 3. Results

### 3.1. Effect of HL on Colonic Crypt Depth in PEDV-Infected Piglets

**Figure 1:**
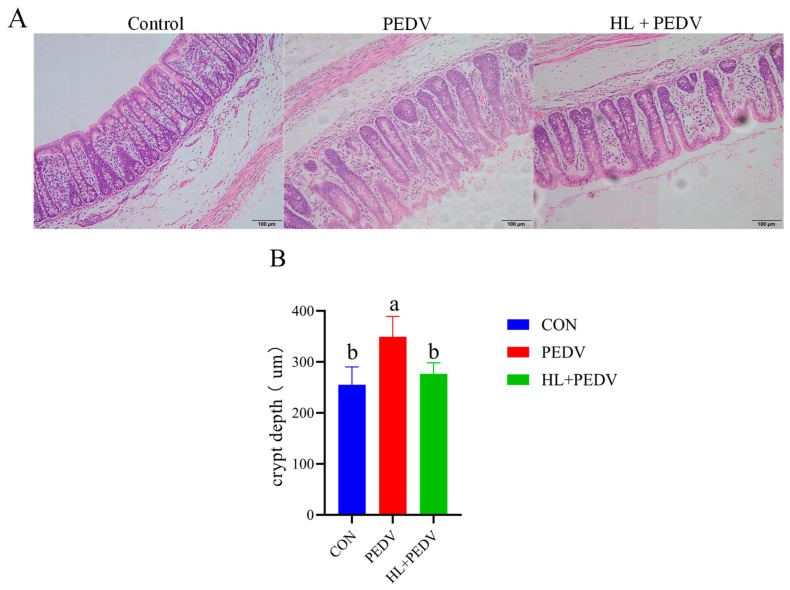
(**A**) Effects of HL supplementation on intestinal morphological structure in PEDV-infected piglets. (**B**) Comparison of crypt depths among CON, PEDV and HL + PEDV groups. CD represents crypt depth. CON, control group; PEDV, PEDV-infected model group; HL + PEDV, HL-treated PEDV-infected group. All data are expressed as mean ± standard error of the mean (n = 6). Means marked with different lowercase superscript letters (a, b) within the same column differ significantly at *p* < 0.05.

As illustrated in Figure 1, the colonic crypt depth was markedly higher (*p* < 0.05) in the PEDV-challenged piglets than that in the control group. Microscopic examination revealed obvious vacuolar degeneration of colonic crypt epithelial cells in PEDV-infected piglets, accompanied by impaired integrity of the intestinal epithelial barrier. Massive exfoliated epithelial debris and inflammatory exudate accumulated in the crypt lumen, while the number of goblet cells was markedly declined. In contrast, dietary HL supplementation significantly decreased colonic crypt depth in PEDV-infected piglets relative to the PEDV alone group (*p* < 0.05). Meanwhile, HL treatment effectively ameliorated epithelial degeneration, necrosis and inflammatory cell infiltration in colonic tissues. Moreover, the accumulation of inflammatory exudate was alleviated, and goblet cell abundance was notably restored in the HL + PEDV group (Figure 1A,B).

### 3.2. Effects of HL on Colonic Antioxidant Capacity in PEDV-Infected Piglets

**Figure 2:**
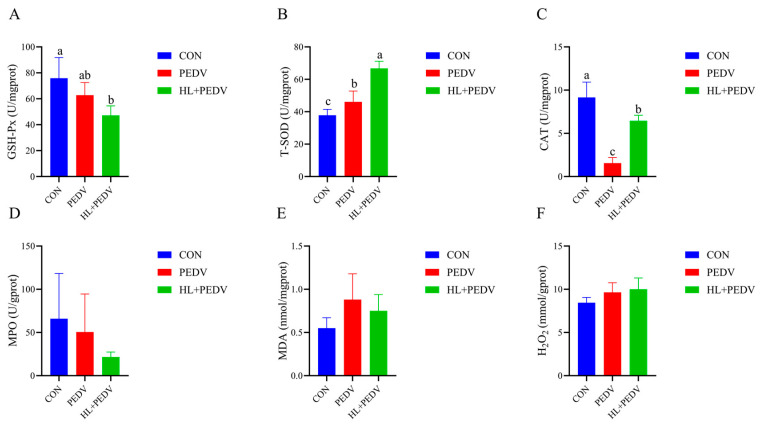
Effects of HL on colonic antioxidant capacity in PEDV-infected piglets. (**A**) The activity of glutathione peroxidase (GSH-Px) in colon tissue; (**B**) Total superoxide dismutase (T-SOD) activity of colonic samples; (**C**) Catalase (CAT) activity in piglet colon; (**D**) Myeloperoxidase (MPO) activity of colonic tissue; (**E**) Malondialdehyde (MDA) content in colon; (**F**) Hydrogen peroxide (H_2_O_2_) concentration of colonic tissue. CON, blank control group; PEDV, PEDV-challenged group; HL + PEDV, HL-supplemented PEDV-challenged group. CON, control group; PEDV, PEDV-infected group; HL + PEDV, HL-treated PEDV-infected group. All data are expressed as mean ± standard error of the mean (n = 6). Bars within the same panel marked with different lowercase superscript letters (a, b, c) are significantly different at *p* < 0.05, while bars sharing the combined superscript “ab” show no significant difference compared with those labeled “a” or “b”.

As shown in Figure 2, relative to the CON group, PEDV challenge markedly decreased (*p* < 0.05) colonic CAT activity in piglets, whereas colonic T-SOD activity was notably elevated following PEDV infection. Compared with the PEDV group, HL supplementation effectively restored (*p* < 0.05) the activities of CAT and T-SOD to a significantly higher level.

### 3.3. Effect of HL on the Expression of PEDV-Related Genes in the Colons of Piglets

**Figure 3:**
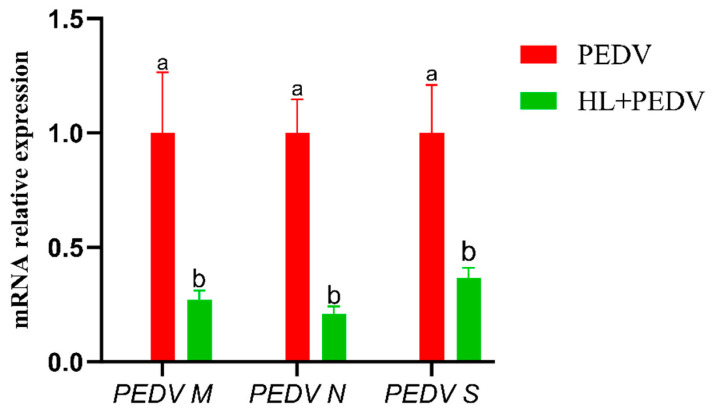
Effect of HL on the Expression of PEDV-Related Genes in the Colons of Piglets. PEDV, PEDV-infected group; HL + PEDV, HL + PEDV-infected group. Data are presented as the mean ± standard error of the mean for each group (n = 6). Means within columns with different lowercase superscript letters (a, b) differ significantly (*p* < 0.05).

As illustrated in Figure 3, the transcriptional levels of *PEDV M*, *N*, and *S* genes in the colons of piglets in the HL + PEDV group were significantly downregulated compared with those in the PEDV group (*p* < 0.05).

### 3.4. Effects of HL on the Expression of Inflammatory and Immune-Related Genes in the Colons of PEDV-Infected Piglets

**Figure 4:**
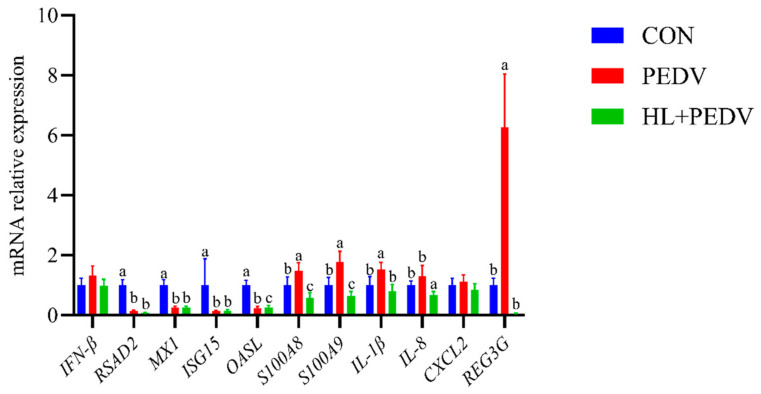
Effects of HL on the expression of inflammatory and immune-related genes in piglets infected with PEDV. *IFN-β*, Interferon Beta; *RSAD2*, Radical S-Adenosyl Methionine Domain Containing 2; *MX1*, MX Dynamin Like GTPase 1; *ISG15*, Interferon Stimulated Gene 15; *OASL*, 2′,5′-Oligoadenylate Synthetase Like; *S100A8*, S100 Calcium Binding Protein A8; *S100A9*, S100 Calcium Binding Protein A9; *IL-1β*, Interleukin 1 Beta; *IL-8*, Interleukin 8; *CXCL2*, C-X-C Motif Chemokine Ligand 2; *REG3G*, Regenerating Family Member 3 Gamma. CON, Control group; PEDV, PEDV-infected group; HL + PEDV, HL + PEDV-infected group. Data are presented as the mean ± standard error of the mean (n = 6). Means within columns with different lowercase superscript letters (a, b, c) differ significantly (*p* < 0.05).

As illustrated in Figure 4, compared with the CON, the transcriptional levels of *RSAD2*, *MX1*, *ISG15*, and *OASL* in the colons of piglets in the PEDV group were significantly decreased (*p* < 0.05), whereas the expression levels of *S100A8*, *S100A9*, *IL-1β*, and *REG3G* were significantly increased (*p* < 0.05). When compared with the PEDV group, the HL + PEDV group showed a significant reduction in the expression levels of the OASL gene as well as inflammatory genes including *S100A8*, *S100A9*, *IL-1β*, *IL-8*, and *REG3G* in the colon tissues (*p* < 0.05).

### 3.5. HL Regulates the Expression of Genes Related to Colonic Tissue Damage and Repair Following PEDV Infection in Piglets

**Figure 5:**
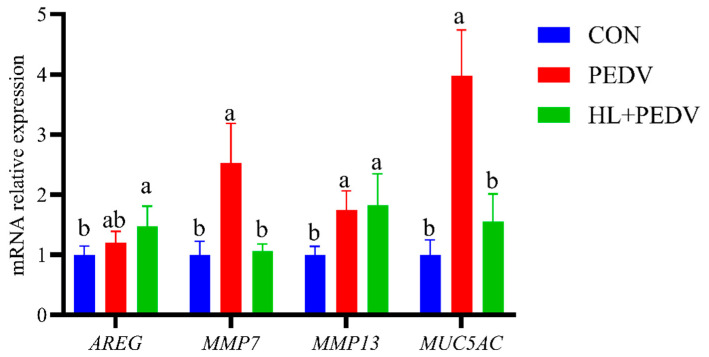
HL alters the expression of genes associated with colonic tissue damage and repair in PEDV-infected piglets. *AREG*: Amphiregulin; *MMP7*: Matrix Metallopeptidase 7; *MMP13*: Matrix Metallopeptidase 13; *MUC5AC*: *Mucin 5AC*, Oligomeric Mucus/Gel-Forming; *IFN-β*: Interferon Beta; *CON*: control group. PEDV: PEDV-infected group. HL + PEDV: HL + PEDV-infected group. Data are presented as the mean ± standard error of the mean (n = 6). Means within columns with different lowercase superscript letters (a, b) differ significantly (*p* < 0.05), while those with the combined superscript letter (ab) exhibit no significant difference compared to groups labeled with a or b alone.

As illustrated in Figure 5, compared with the CON, the relative mRNA expression levels of *MMP7*, *MMP13*, and *MUC5AC* in the colons of piglets in the PEDV group were significantly upregulated (*p* < 0.05). Following the HL feeding intervention in PEDV-infected piglets, the transcriptional levels of *MMP7* and *MUC5AC* in the colonic tissues were significantly decreased compared with those in the PEDV group (*p* < 0.05).

### 3.6. Effects of HL on the Expression Levels of Genes Associated with Water and Ion Transport Channels in the Colons of Piglets Infected with PEDV

**Figure 6:**
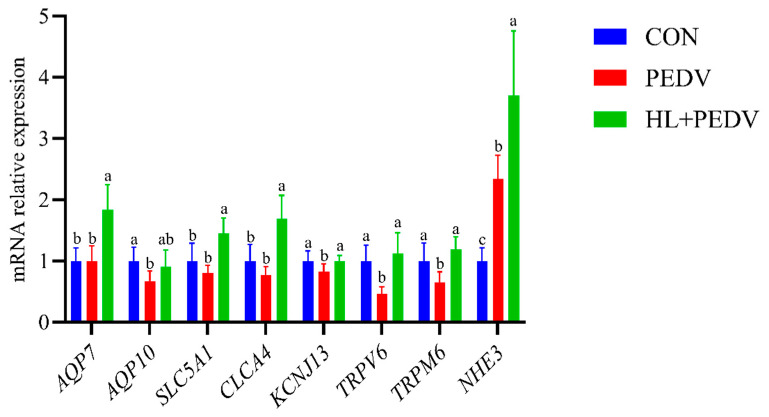
Effects of HL on the expression levels of genes associated with water and ion transport channels in the colon in PEDV-infected piglets. *AQP7*, Aquaporin 7; *AQP10*, Aquaporin 10; *SLC5A1*, Solute Carrier Family 5 Member 1; *CLCA4*, Chloride Channel Accessory 4; *KCNJ13*, Potassium Inwardly Rectifying Channel Subfamily J Member 13; *TRPM6*, Transient Receptor Potential Cation Channel Subfamily M Member 6; *TRPV6*, Transient Receptor Potential Cation Channel Subfamily V Member 6; *NHE3*, Sodium Hydrogen Exchanger 3; CON: Control group. PEDV: PEDV-infected group. HL + PEDV: HL + PEDV-infected group. Data are presented as the mean ± standard error of the mean for each group (n = 6). Values within each panel bearing different lowercase superscript letters (a, b, c) are significantly different (*p* < 0.05). Samples marked with the shared combined superscript (ab) have no statistical difference versus groups only labeled a or b.

As illustrated in Figure 6, compared with the CON, the mRNA expression levels of *AQP10* and *TRPV6* in the colons of piglets were significantly downregulated after PEDV infection (*p* < 0.05), whereas the mRNA expression level of *NHE3* was significantly upregulated (*p* < 0.05). In comparison with the PEDV group, the mRNA expression levels of *AQP7*, *CLCA4*, and *NHE3* in the colons of piglets in the HL + PEDV group were significantly increased (*p* < 0.05).

## 4. Discussion

As a key component of the large intestine in piglets, the colon plays a key role in physiological processes such as water and electrolyte absorption, maintenance of the intestinal barrier [15], and immune defense. The underlying cause of diarrhea in piglets is an imbalance in the absorption and secretion of intestinal fluids and electrolytes. PEDV infection can disrupt the intestinal epithelial barrier, leading to microbial imbalance, weakened intestinal immunity, and severe diarrhea [16,17,18,19]. The unique crypt structure of the piglet intestinal mucosa serves as a crucial structural foundation for intestinal epithelial regeneration and nutrient absorption; changes in crypt depth can directly reflect the extent of intestinal mucosal damage and the repair process [20,21]. Abnormal differentiation of goblet cells within the crypts and reduced mucus secretion further exacerbate damage to the intestinal mucosa [6].

The results of this study show that colonic crypt depth was significantly increased in the PEDV group, with epithelial cells exhibiting marked vacuolar degeneration, necrosis, and desquamation; inflammatory cell infiltration in the lamina propria was significantly exacerbated; and the number of goblet cells was significantly reduced [22,23,24]. A previous study showed that PEDV infection significantly increases colonic crypt depth in piglets [25], suggesting that after the virus causes intestinal mucosal damage, the body initiates a mucosal repair process by enhancing epithelial cell proliferation, which is consistent with the phenomenon of colonic crypt hyperplasia observed in PEDV-infected piglets in the present study. Another study revealed that PEDV infection downregulates goblet cell differentiation by activating the Notch signaling pathway (specifically via the viral ORF3 protein enhancing the expression of Notch ligands JAG-1/DLL4), leading to a reduction in the number of goblet cells in the intestines of newborn piglets and impaired intestinal barrier integrity [6]. The findings of these two studies strongly support the conclusions of the present study regarding PEDV-induced colonic morphological damage. In response to the deepening of colonic crypts, epithelial cell damage, and disruption of intestinal structure caused by PEDV infection, this study found that HL intervention effectively reversed these pathological changes, significantly alleviating the degree of colonic crypt deepening, reducing epithelial cell vacuolization, necrosis, and desquamation, and restoring the integrity of the intestinal mucosal structure. This result is consistent with previous studies on the inhibitory effects of Chebulinic acid against PEDV infection [9].

Following PEDV infection, piglets experience severe oxidative stress characterized by disrupted redox balance [26]. This manifests as mitochondrial dysfunction with massive ROS accumulation and suppressed antioxidant defenses, leading to elevated lipid peroxidation products [27,28]. Abnormal ROS accumulation induces lipid peroxidation and oxidative DNA damage, damaging cellular structure and function [29]. Oxidative stress causes direct damage to intestinal and hepatic tissues while activating Z-RNA/ZBP1 pathways [30]. The ROS/HIF-1α axis reprograms metabolism to support viral replication and compromises intestinal barrier function [31,32]. Supplementation with tannic acid-zinc has shown potential to alleviate intestinal injury [33], and the body modulates antioxidant enzymes such as CAT and GPX4 [28]. The results of this study show that GSH-Px activity in the colons of piglets in the PEDV group was significantly reduced, indicating that PEDV infection induced oxidative stress in the piglets. PEDV infection significantly reduced CAT activity in the piglets’ colons, suggesting that viral infection disrupted the redox balance in colonic tissue, inducing significant oxidative stress damage. Following HL treatment, CAT activity in the piglets’ colons significantly recovered, and the degree of oxidative stress was markedly alleviated, suggesting that HL can mitigate PEDV-induced colonic oxidative stress damage and maintain normal physiological metabolism in the colon by regulating the function of the antioxidant enzyme system. These findings are consistent with the conclusions of previous research [13], whose study demonstrated that *Terminalia chebula* extract can enhance the antioxidant capacity of weaned piglets, improve gut health, and reduce oxidative stress damage. The mechanism may be related to HL’s activation of the body’s antioxidant pathways and promotion of antioxidant enzyme synthesis, which is consistent with the known antioxidant properties of *Terminalia chebula* and its extracts [8].

PEDV contains three key structural genes: *M*, *N*, and *S* [34,35,36,37]. The *M* gene sequence is highly conserved and encodes a viral membrane protein that maintains the stability of the viral envelope, participates in viral assembly and progeny virus release, and regulates the host’s innate immune response. The *PEDV M* protein interacts with the inhibitory domain of IRF7, inhibiting TBK1/IKKε-induced IRF7 phosphorylation and dimerization, thereby suppressing type I interferon production [34]. The *N* gene is highly stable and encodes the nucleocapsid protein, which encapsulates the viral RNA, protects the viral genome, regulates viral replication and transcription, and is a common target for clinical detection. The *PEDV N* protein antagonizes β-interferon production by blocking the interaction between TBK1 and IRF3, evading the host’s innate immune response [35]. Furthermore, the *N* gene downregulates HDAC1 expression, inducing STAT1 acetylation rather than phosphorylation, which blocks STAT1 nuclear translocation and inhibits interferon-stimulated gene expression, aiding immune evasion [36]. The *S* gene exhibits high variability; it encodes the spike protein, which recognizes and binds to host cell receptors to mediate viral entry and infection, and is a key gene for viral strain typing and vaccine development [37,38]. The mRNA expression levels of these three genes directly reflect the virus’s replication capacity in colonic tissue [39].

This study found that HL treatment significantly downregulated the transcriptional levels of the *M*, *N*, and *S* genes in the colons of piglets in the PEDV group, indicating that HL can effectively inhibit viral replication in colonic tissue, alleviate persistent damage to the intestinal mucosa caused by the virus, and exert a direct antiviral effect. These findings suggest that HL may inhibit PEDV proliferation by regulating viral replication-related signaling pathways within host cells or by enhancing the host’s innate immune response, providing a theoretical basis for its use as a nutritional intervention to prevent and control PEDV infection. These findings align with previous research [40] on PEDV suppression by natural products and are partially consistent with findings on *Terminalia chebula*’s pharmacological properties [8] and its extract’s immune-enhancing effects on intestinal health [41].

Abnormal activation of the intestinal inflammatory response is another key mechanism by which PEDV infection causes colonic damage. *S100A8* and *S100A9* are calcium-binding proteins that induce cytokine expression and regulate the NLRP3 inflammasome [42,43]. *REG3G* is an antimicrobial peptide that participates in intestinal barrier defense and connects the intestinal microbiome with host physiology [44]. PEDV infection has been shown to activate the NLRP3 inflammasome to mediate interleukin-1β secretion in porcine intestinal epithelial cells [45], indicating that viral infection can induce a severe intestinal inflammatory response. The expression levels of interferon-stimulated genes *RSAD2*, *MX1* and *ISG15* were markedly decreased in the PEDV compared with the control group. This finding indicates that PEDV can exert immune evasion capability and effectively suppress the host interferon-stimulated antiviral pathways to facilitate viral proliferation [46,47,48]. Furthermore, HL treatment failed to reverse the downregulation of these three antiviral genes under the present experimental conditions, suggesting that HL cannot modulate or rescue the impaired interferon-related antiviral response induced by PEDV. HL intervention significantly reduced the transcriptional levels of the aforementioned inflammation-related genes, indicating that HL can alleviate PEDV-induced colonic inflammatory damage by inhibiting the excessive activation of inflammatory signaling pathways; this is consistent with the findings of relevant previous studies [13], whose study demonstrated that *Terminalia chebula* extract enhances anti-inflammatory capacity and improves gut health in weaned piglets by regulating cytokine secretion.

*MMP7* and *MMP13* belong to the matrix metalloproteinase family and are key genes involved in intestinal tissue repair and remodeling; abnormally elevated expression of these genes indicates excessive tissue remodeling, which can exacerbate intestinal mucosal damage [49,50]. *MUC2* and *MUC5AC* are the primary mucins constituting the intestinal mucus barrier [51]. Among these, *MUC2* is a core structural component of the mucus barrier, and its downregulation directly impairs mucus barrier function [52]. This phenomenon was also confirmed by relevant previous studies [6], who found that PEDV infection inhibits *MUC2* secretion by suppressing goblet cell differentiation. In contrast, *MUC5AC* is expressed at extremely low levels in healthy intestines, and its abnormal upregulation typically indicates that the intestine is in a pathological repair state [53]. This study found that PEDV infection significantly downregulates *MUC2* expression while simultaneously upregulating the expression of *MMP7*, *MMP13*, and *MUC5AC*. This indicates that viral infection disrupts the integrity of the colonic mucus barrier and tissue homeostasis, leading to impaired intestinal barrier function, which is consistent with the findings observed by previous research [52]. HL intervention significantly reduced the abnormally high expression of *MMP7*, *MMP13*, and *MUC5AC*, alleviating excessive tissue damage, and also synergistically promoted colonic tissue repair and functional restoration. This is consistent with the findings of previous research [41] regarding the ability of Chebulic myrobalan extract to improve gut health and enhance intestinal barrier function [8]. This demonstrates that HL possesses the potential efficacy to repair intestinal damage by regulating the expression of mucus barrier-related genes.

This study also investigated transmembrane transport of water and ions in piglets’ intestines, a core mechanism for maintaining intestinal health. For example, *CLCA4* is a calcium-activated chloride channel regulatory gene highly expressed in small intestine, lung, and colon tissues [54]. Research indicates that *CLCA4* plays a role in various intestinal diseases and is associated with the development of multiple cancers, such as esophageal cancer and colorectal cancer; its expression can significantly inhibit tumor cell growth [55,56,57], and is particularly important for the physiological health of piglets’ intestines. *NHE3* is a key gene that regulates cellular acid-base balance through Na^+^/H^+^ exchange and promotes sodium absorption in the gut, playing a crucial role in intestinal health [58].

This study found that PEDV significantly reduced the intestinal mRNA expression of *AQP10* and *TRPV6* and decreased the mRNA expression levels of *AQP7* and *TRPM6*. The findings of previous research [59], which showed that PEDV infection leads to downregulation of *TRPV6* expression, are consistent with the conclusions of this study, indicating that the inhibitory effect of PEDV on intestinal calcium channels is confirmed. The findings of previous research [60], which reported that PEDV significantly reduced the intestinal mRNA expression of *AQP10*, *AQP7*, *TRPV6*, and *TRPM6*, are consistent with the conclusions of this study, further confirming the extensive inhibitory effect of PEDV on the expression of intestinal water channels and ion channels. In contrast, HL significantly increased the mRNA expression levels of *AQP7*, *CLCA4*, and *NHE3* in the colons of PEDV-infected piglets, while simultaneously upregulating the transcriptional levels of *AQP10*, *TRPV6*, and *TRPM6*. This improved the transmembrane transport capacity of Ca^2+^ and Mg^2+^ in the colonic mucosa, restored intestinal ion balance, and facilitated the maintenance of normal intestinal physiological functions. This indicates that HL restores the homeostasis of the intestinal microenvironment by regulating the expression of aquaporins and ion transporters, thereby providing a new therapeutic target for the prevention and control of PEDV infection.

This study demonstrates that HL can protect the colonic structure and function of PEDV-infected piglets through multiple synergistic pathways. The primary mechanisms include regulating colonic crypt depth to restore intestinal morphological integrity, modulating the antioxidant enzyme system to alleviate oxidative stress damage, inhibiting the expression of PEDV structural proteins to reduce viral replication, downregulating the expression of inflammation-related genes to mitigate excessive inflammatory damage, and regulating the expression of tissue repair-related genes and ion transport genes to promote colonic structural repair and maintain functional homeostasis. These findings systematically elucidate the molecular mechanisms by which HL, as a nutritional intervention, alleviates PEDV-induced colonic damage in piglets. They provide new insights and theoretical support for the prevention and control of PEDV infection in swine production, while also offering more robust scientific evidence for the application of HL in the regulation of intestinal health.

This study has an obvious limitation of small sample size, with only six piglets in each group and equal numbers of male and female individuals. The small sample reduces statistical power and weakens the confidence of molecular indicator interpretation. Meanwhile, we cannot evaluate sex effects and treatment-sex interactions due to insufficient animal numbers. Subsequent trials will expand sample capacity to further verify the present findings.

## 5. Conclusions

The colonic damage induced by PEDV is a major cause of severe diarrhea and high mortality in piglets, posing a substantial economic threat to the global swine industry. The present study indicated that HL supplementation exerts beneficial effects on alle-viating PEDV-induced colonic injury through multiple potential regulatory mechanisms, improving abnormal colonic histological structure, downregulating the transcription of PEDV structural genes, and mitigating excessive inflammatory responses. This work illustrates that HL supplementation can relieve PEDV-triggered colonic damage via multiple synergistic regulatory pathways: improving abnormal crypt morphology, reducing the transcriptional level of PEDV related genes, suppressing excessive inflammatory response, and stabilizing colonic ion transport function. HL treatment modulates the key pathological processes above and improves colonic biochemical and molecular phenotypes, showing certain protective effects against PEDV-associated intestinal damage in piglets. This study provides a preliminary scientific reference for the application of HL as a potential nutritional intervention strategy to relieve PEDV-induced intestinal injury in piglets.

## Figures and Tables

**Table 1:** Primer Sequences.

Gene Name	Forward Primer (5′-3′)	Reverse Primer (5′-3′)	Accession Number
*AREG*	GAGTACGATAACGAACCGCACA	TTTCCACTTTTGCCTCCCTTT	NM_214376
*CXCL2*	CGGAAGTCATAGCCACTCTCAA	CAGTAGCCAGTAAGTTTCCTCCATC	NM_001001861
*GPX2*	CCGGGACTTCACCCAACTC	CGGACGTACTTGAGGCTGTT	NM_001115136
*IL-1β*	CAACGTGCAGTCTATGGAGT	GAGGTGCTGATGTACCAGTTG	NM_214055
*IL-8*	TTCGATGCCAGTGCATAAATA	CTGTACAACCTTCTGCACCCA	NM_213867
*MMP13*	AGTTTGGCCATTCCTTAGGTCTTG	GGCTTTTGCCAGTGTAGGTATAGAT	XM_003129808
*MMP7*	GGTGGCAGCATAGGCATTAAC	TCCGTAGGTTGGATACATCACAG	NM_001348795
*MUC5AC*	GTCAATGGCCGCACAATTCAG	CATCGTGGGAGAGGAACTCG	XM_021082583
*OASL*	GGCACCCCTGTTTTCCTCT	AGCACCGCTTTTGGATGG	NM_001031790
*M*	TCCCGTTGATGAGGTGAT	AGGATGCTGAAAGCGAAAA	KT021228
*N*	TTGGTGGTAATGTGGCTGTTC	TGGTTTCACGCTTGTTCTTCTT	KT021228
*S*	CTCTCTGGTACAGGCAGCAC	GCTCACGTAGAGTCAAGGCA	KT021228
*RSAD2*	CCCCACTAGCGTCAATTACC	TGATCTTCTCCATACCCGCT	NM_213817
*REG3G*	CTGTCTCAGGTCCAAGGTGAAG	CAAGGCATAGCAGTAGGAAGCA	XM_005662419
*S100A8*	AACTCTGTTTCGGGGAGACC	CGCGTAGATGGCGTGGTAA	NM_001160271
*S100A9*	CCAGGATGTGGTTTATGGCTTTC	CGGACCAAATGTCGCAGA	XM_013997035
*TRPM6*	TACGGGAAGAGATGTGGTGT	CGCCTGAGCTTCATCTCATT	XM_021064975
*TRPV6*	AGGAGCTGGTGAGCCTCAAGT	GGGGTCAGTTTGGTTGTTGG	NM_001436069

AREG, Amphiregulin; CXCL2, C-X-C motif chemokine ligand 2; GPX2, Glutathione peroxidase 2; IL-1β, Interleukin-1 beta; IL-8, Interleukin-8; MMP13, Matrix metalloproteinase 13; MMP7, Matrix metalloproteinase 7; MUC5AC, Mucin 5AC, oligomeric mucus/gel-forming; OASL, 2′-5′-oligoadenylate synthetase like, M, Porcine epidemic diarrhea virus M gene; N, Porcine epidemic diarrhea virus N gene; S, Porcine epidemic diarrhea virus S gene; RSAD2, Radical S-adenosyl methionine domain containing 2; REG3G, Regenerating family member 3 gamma; S100A8, S100 calcium binding protein A8; S100A9, S100 calcium binding protein A9; TRPV6, Transient receptor potential vanilloid 6; TRPM6, Transient receptor potential melastatin 6.

## Data Availability

All data presented in this research is included in the article. Further inquiries can be directed at the corresponding author.
